# Delinking CARD9 and IL-17: CARD9 Protects against *Candida tropicalis* Infection through a TNF-α–Dependent, IL-17–Independent Mechanism

**DOI:** 10.4049/jimmunol.1500870

**Published:** 2015-09-02

**Authors:** Natasha Whibley, Jillian R. Jaycox, Delyth Reid, Abhishek V. Garg, Julie A. Taylor, Cornelius J. Clancy, M. Hong Nguyen, Partha S. Biswas, Mandy J. McGeachy, Gordon D. Brown, Sarah L. Gaffen

**Affiliations:** * Division of Rheumatology and Clinical Immunology, Department of Medicine, University of Pittsburgh, Pittsburgh, PA 15261;; † Department of Biological Sciences, Carnegie Mellon University, Pittsburgh, PA 15213;; ‡ Aberdeen Fungal Group, Division of Applied Medicine, Immunity, Infection and Inflammation Programme, University of Aberdeen, Aberdeen AB25 2ZD, United Kingdom; and; § Division of Infectious Diseases, Department of Medicine, University of Pittsburgh, Pittsburgh, PA 15261

## Abstract

*Candida* is the third most common cause of bloodstream infections in hospitalized patients. Immunity to *C. albicans*, the most frequent species to be isolated in candidiasis, involves a well-characterized Dectin-1/caspase-associated recruitment domain adaptor 9 (CARD9)/IL-17 signaling axis. Infections caused by non-*albicans Candida* species are on the rise, but surprisingly little is known about immunity to these pathogens. In this study, we evaluated a systemic infection model of *C. tropicalis*, a clinically relevant, but poorly understood, non-*albicans Candida*. Mice lacking CARD9 were profoundly susceptible to *C. tropicalis*, displaying elevated fungal burdens in visceral organs and increased mortality compared with wild-type (WT) controls. Unlike *C. albicans*, IL-17 responses were induced normally in CARD9^−/−^ mice following *C. tropicalis* infection. Moreover, there was no difference in susceptibility to *C. tropicalis* infection between WT and IL-23p19^−/−^, IL-17RA^−/−^, or Act1^−/−^ mice. However, TNF-α expression was markedly impaired in CARD9^−/−^ mice. Consistently, WT mice depleted of TNF-α were more susceptible to *C. tropicalis*, and CARD9-deficient neutrophils and monocytes failed to produce TNF-α following stimulation with *C. tropicalis* Ags. Both neutrophils and monocytes were necessary for defense against *C. tropicalis*, because their depletion in WT mice enhanced susceptibility to *C. tropicalis*. Disease in CARD9^−/−^ mice was not due to defective neutrophil or monocyte recruitment to infected kidneys. However, TNF-α treatment of neutrophils in vitro enhanced their ability to kill *C. tropicalis*. Thus, protection against systemic *C. tropicalis* infection requires CARD9 and TNF-α, but not IL-17, signaling. Moreover, CARD9-dependent production of TNF-α enhances the candidacidal capacity of neutrophils, limiting fungal disease during disseminated *C. tropicalis* infection.

## Introduction

Fungal infections are an escalating problem worldwide, yet fungi remain remarkably understudied compared with bacteria and viruses (1). Infections caused by *Candida* spp. range from superficial mucocutaneous infections to invasive organ infections and disseminated candidiasis. Disseminated candidiasis is a particularly significant problem in hospital settings, and *Candida* is the most common fungal microbe and the third most common pathogen responsible for nosocomial bloodstream infections (2). Increasing drug resistance, a lack of antifungal vaccines, and high mortality of up to 80% highlight a compelling need for alternative or complementary treatments (2, 3).

Our understanding of immunity to *Candida* infections is largely based on studies of the most common species, *C. albicans.* Antifungal immunity to *C. albicans* has been the subject of numerous studies, and a detailed picture of the essential components has emerged, involving a Dectin/CARD9/IL-17 pathway (4). *C. albicans* is sensed by C-type lectin receptors (CLRs) including Dectin-1, Dectin-2, and Dectin-3 (5). CLRs signal through the adaptor caspase-associated recruitment domain adaptor 9 (CARD9), and CARD9^−/−^ mice are profoundly susceptible to disseminated *C. albicans* infection (6). Consistently, patients with mutations in CARD9 are susceptible to mucocutaneous and disseminated candidiasis (7–9). CARD9 is primarily expressed in myeloid cells, including neutrophils, macrophages, and dendritic cells, although low expression of CARD9 was observed in other cell types, including T and NK cells (10, 11). Upon receptor activation, CARD9 forms a signaling complex with BCL10 and MALT1 to activate NF-κB and induce cytokines, including IL-6, IL-1β, IL-23, and TNF-α (4, 12). Notably, many of these cytokines are inductive for IL-17/Th17 responses; accordingly, CARD9^−/−^ splenocytes were shown to be defective in IL-17A production in response to *C. albicans* stimulation (13).

IL-17 signaling is required for protection against disseminated *C. albicans* infection. IL-17RA^−/−^, RORγt^−/−^, and IL-17A^−/−^ mice are susceptible to disseminated candidiasis (14–17). Thus, the mechanism by which CARD9 exerts its protective effects during disseminated candidiasis is believed to be the induction of a protective IL-17/Th17 response. However, CARD9 also mediates in other antifungal activities, such as reactive oxygen species production and neutrophil killing, which are also important in combating fungal pathogens (18–20).

Although *C. albicans* remains the dominant disease-causing pathogen of this genus, rates of infections caused by non-*albicans Candida* (NAC) species are increasing (21); the most commonly isolated are *C. tropicalis*, *C. glabrata*, *C. parapsilosis*, and *C. krusei*. NAC species account for ≥50% of candidemia cases (22). Of concern, mortality associated with invasive infections caused by NAC species is higher than that caused by *C. albicans* (23). *C. tropicalis* is particularly associated with invasive infection, accounting for up to 45% of NAC infections (22, 24). Moreover, higher mortality with disseminated *C. tropicalis* compared with *C. albicans* infection was documented in patients with acute leukemia or those undergoing bone marrow transplantation, for whom neutropenia appears to be a particularly important risk factor (22, 25–27). In mice, neutropenia following cyclophosphamide or cytarabine treatment increases susceptibility to systemic *C. tropicalis* infection (28, 29). However, surprisingly little is known regarding immunity to disseminated *C. tropicalis* infection.

Although *C. tropicalis* and *C. albicans* are related phylogenetically (30), important differences exist between these species. *C. albicans* and *C. tropicalis* differ in their metabolic capacities: only *C. tropicalis* can ferment sucrose (31, 32). Additionally, *C. albicans* and *C. tropicalis* differ in their expression of secreted aspartic proteinases, important virulence factors (33, 34). Thus, despite their evolutionary relationship, it cannot be assumed that host immunity to *C. tropicalis* mirrors that of *C. albicans*.

The increase in NAC infections highlights a need to understand immunity to these emerging pathogens. In this study, we investigated the factors responsible for antifungal immunity against a clinically relevant NAC species, *C. tropicalis*. Using a murine model of disseminated infection, we demonstrated that CARD9 was crucial for host defense against *C. tropicalis*. Surprisingly, protection against *C. tropicalis* infection was not IL-17 dependent. However, TNF-α blockade caused increased susceptibility to infection, and TNF-α responses were impaired in infected CARD9^−/−^ mice. TNF-α treatment enhanced the killing capacity of neutrophils against *C. tropicalis*, suggesting that CARD9-dependent TNF-α responses are an important mechanism to control *C. tropicalis* growth during disseminated infection.

## Materials and Methods

### Mice

Wild-type (WT) mice (C57BL/6) were from The Jackson Laboratory (Bar Harbor, ME). Rag2^−/−^ and Rag2^−/−^Il2rg^−/−^ mice were from Taconic Farms. CARD9^−/−^ and Act1^−/−^ (also known as CIKS^−/−^) mice were generated as described (11, 35). IL-17RA^−/−^ mice were from Amgen, and IL-23p19^−/−^ mice were from Genentech. Dectin-1^−/−^ and Dectin-2^−/−^ mice on a C57BL/6 background were generated as described (36, 37). All experiments included age- and sex-matched controls on the C57BL/6 background. Protocols were approved by the University of Pittsburgh Institutional Animal Care and Use Committee or the University of Aberdeen Animal Welfare and Ethical Review Body and the UK Home Office and adhered to guidelines in the *Guide for the Care and Use of Laboratory Animals* of the National Institutes of Health.

### *C. tropicalis* culture and disseminated infection

An isolate of *C. tropicalis* (W4162870) recovered from a patient with candidemia was used in all experiments. *C. tropicalis* was grown in yeast extract peptone dextrose at 30°C for 18–24 h. Mice were injected via the tail vein with 100 μl sterile saline or 1 × 10^4^ CFU/g *C. tropicalis* yeast cells. Mice were weighed and monitored daily and sacrificed if they showed >20% weight loss or signs of severe pain or distress. At sacrifice, organs were weighed and homogenized in sterile PBS using a GentleMACS (Miltenyi Biotec, Cambridge, MA). Serial dilutions of organ homogenates were plated on yeast extract peptone dextrose agar with antibiotics, and fungal burdens were determined by CFU enumeration. For TNF-α blockade, mice were injected i.p. with 100 μg etanercept (Amgen, Thousand Oaks, CA) every other day starting at day −1 (continuous treatment) or day 2 or 5 (delayed treatment). PBS was used as a control.

### Neutrophil and monocyte depletion

Mice were injected i.p. with Abs on days −1 and +1 postinfection. Anti-Ly6G (clone 1A8) and IgG2a isotype control (clone 2A3) were injected at a dose of 300 μg. Anti-Gr1 (clone RB6-8C5) and IgG2b isotype control (clone LTF-2) were injected at a dose of 80 μg. All Abs were from Bio X Cell (West Lebanon, NH). Alternatively, mice were injected i.v. with 200 μl PBS, PBS liposomes, or clodronate liposomes (clodronateliposomes.com, Amsterdam, The Netherlands) 1 or 3 d prior to infection. To assess neutrophil and monocyte depletion, blood was collected from the tail vein immediately before *C. albicans* infection on day 0, and cell populations were enumerated by flow cytometry.

### Quantitative PCR

At sacrifice, kidneys and spleens were stored at −80°C. Frozen tissues were lysed on a GentleMACS (Miltenyi Biotec), and total RNA was extracted with RNeasy Mini Kits (Qiagen). cDNA was synthesized with SuperScript III First-Strand (Invitrogen). Gene expression was determined by quantitative PCR with PerfeCTa SYBR Green FastMix ROX (Quanta BioSciences) on a 7300 Real-Time PCR System (Applied Biosystems). Primers were from Quantitect (Qiagen). Samples were plated in triplicate and normalized to *Gapdh*.

### Flow cytometry and histology

Blood was collected from tail vein or by cardiac puncture and added to FACS buffer (PBS/1% FBS/2 mM EDTA). RBCs were lysed using 0.2% NaCl, followed by 1.6% NaCl. Kidneys were harvested following perfusion with PBS. Briefly, kidney homogenates were digested in HBSS with 2 mg/ml collagenase type I (Worthington, Lakewood, NJ) and DNase-1 (MP Biochemicals, Solon, OH) for 30 min at 37°C. Kidney cell suspensions were passed through a 40-μm filter and overlaid on Lympholyte-M (CEDARLANE, Burlington, NC). Cells were stained with an Aqua Live/Dead fluorescent dye (Molecular Probes, Eugene OR) and blocked with rat anti-mouse CD16/32 (eBioscience). Cells were stained with the following Abs (from BD Biosciences unless noted): Ly6G-PE or -FITC (clone 1A8), Ly6C-PerCP-Cy5.5 (clone AL-21), CD11b-BV421 or -allophycocyanin (clone M1/70), CD45-FITC (clone 30-F11), and TNF-α–allophycocyanin (clone MP6-XT22; eBioscience). Samples were acquired on a FACS ARIA II or Fortessa (BD Biosciences) and analyzed with FlowJo software (TreeStar).

For histology, kidneys were fixed in 10% formalin, embedded in paraffin, and processed for staining with H&E or periodic acid–Schiff (PAS) with Light Green SF Yellowish counterstain for collagen (Sigma). Images were taken with an EVOS FL Auto microscope (Life Technologies).

### Cell stimulation in vitro and ELISA

Neutrophils and monocytes were isolated from bone marrow cells with a MACS neutrophil or monocyte isolation kit (Miltenyi Biotec), respectively. Neutrophils were resuspended in complete RPMI 1640 (penicillin/streptomycin, l-glutamine, and HEPES) supplemented with 0.5% BSA. Monocytes were resuspended in complete RPMI 1640 containing 5% FBS. A total of 1 × 10^6^ cells/ml was treated or not with heat-killed *C. tropicalis* (HK *C.t*) for 24 h. TNF-α was measured by ELISA in conditioned supernatants (eBioscience). Blood cells were treated or not with HK *C.t* with Golgi Plug (BD Biosciences) for 3 h, and TNF-α was measured by flow cytometry.

### Neutrophil and monocyte killing

Neutrophils and monocytes from bone marrow or blood were plated at 1 × 10^5^ cells/well. Where indicated, cells were preincubated with 20 ng/ml TNF-α (PeproTech, Rocky Hill, NJ) for 1 h. *C. tropicalis* was added to neutrophils or monocytes at 0.5 × 10^5^ yeast cells/well (ratio of 2:1). If indicated, TNF-α was added to *C. tropicalis* before plating. Cultures were incubated with *C. tropicalis* for 2 h and lysed in cold double-distilled H_2_O. Killing was assessed by CFU enumeration in triplicate.

### Statistics

Data were analyzed with Excel and GraphPad Prism (La Jolla, CA) using the log-rank (Mantel–Cox) test, unpaired Student *t* test, Student *t* test with Mann–Whitney correction, or Kruskal–Wallis and post hoc Dunn multiple-comparisons test. The *p* values <0.05 were considered significant.

## Results

### CARD9 is crucial for protection against disseminated *C. tropicalis* infection

**FIGURE 1.**
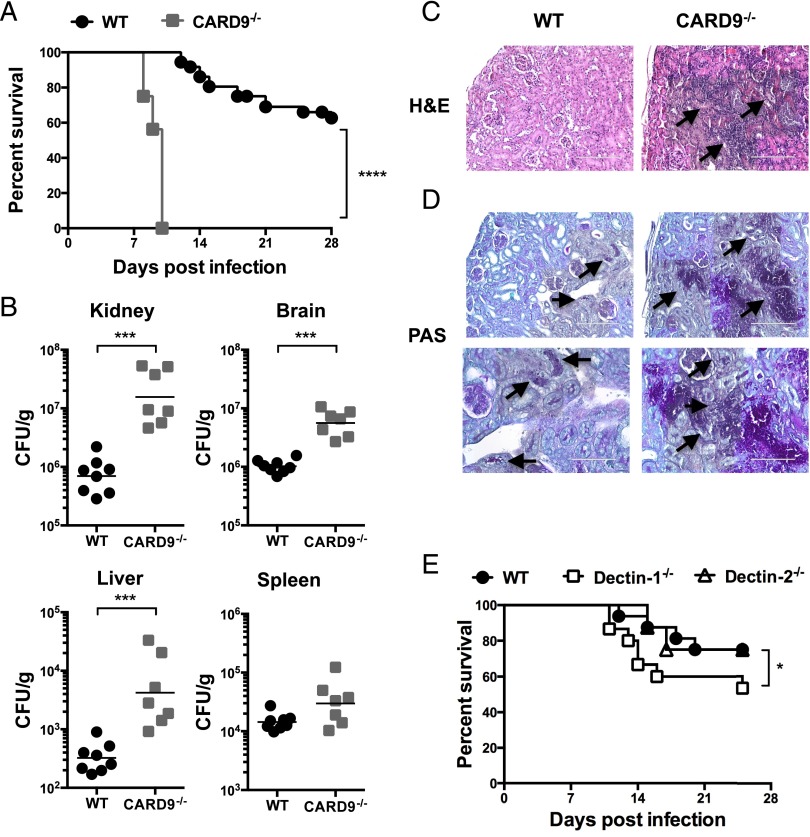
CARD9 is crucial for protection against disseminated *C. tropicalis* infection. WT and CARD9^−/−^ mice were infected i.v. with 1 × 10^4^ CFU/g *C. tropicalis* yeast cells. (**A**) Survival was monitored for 28 d postinfection. Data are pooled from six experiments (WT, *n* = 36; CARD9^−/−^, *n* = 16). ****p < 0.0001, log-rank (Mantel–Cox) test. (**B**) Organ fungal burdens were measured at day 5. Data are pooled from two experiments (each data point represents an individual mouse). ***p < 0.001, Mann-Whitney *U* test. Kidneys isolated from day 5–infected mice were stained with H&E (**C**) or PAS (**D**). Arrows indicate immune cell infiltrate (H&E) and *C. tropicalis* cells (PAS). Scale bars, 200 μm. (**E**) WT, Dectin-2^−/−^, and Dectin-1^−/−^ mice were infected with 1 × 10^4^ CFU/g *C. tropicalis* yeast cells, and survival was monitored for 28 d. Data are pooled from two experiments (WT, *n* = 16; Dectin1^−/−^, *n* = 15; Dectin-2^−/−^, *n* = 8). *p < 0.05, log-rank (Mantel–Cox) test.

To determine whether CARD9 is required for protection against disseminated *C. tropicalis* infection, WT and CARD9^−/−^ mice were infected i.v. with 1 × 10^4^ CFU/g *C. tropicalis* yeast cells, and survival and fungal burden were assessed. With this infective dose, ∼40% mortality was observed in WT mice at day 28 postinfection (Fig. 1A). In contrast, CARD9^−/−^ mice infected with *C. tropicalis* showed 100% mortality by day 10 postinfection, a time point at which all WT mice remained alive (Fig. 1A). Consistent with their early mortality, CARD9^−/−^ mice exhibited higher fungal burdens in visceral organs (kidneys, brain, and liver) than did WT mice, which was measured at day 5 when all mice were still viable (Fig. 1B). There was no difference in CFU in spleens of WT and CARD9^−/−^ mice (Fig. 1B), suggesting tissue-specific differences in *C. tropicalis* infection.

The kidneys are a primary target organ in human candidiasis, because 30–50% of mortality is due to renal insufficiency (38). Tropism to kidney has similarly been demonstrated in mice during disseminated *C. tropicalis* infection (29). Therefore, we evaluated kidneys from *C. tropicalis*–infected WT and CARD9^−/−^ mice on day 5 by histology (H&E staining to assess immune infiltrates and PAS staining to visualize fungi). Immune cells were seen in kidneys of both WT and CARD9^−/−^ mice, although more infiltration was evident in CARD9^−/−^ mice (Fig. 1C). *C. tropicalis* was more prominent and more dispersed throughout the kidney cortex in CARD9^−/−^ mice compared with WT mice, suggestive of a failure to restrain *C. tropicalis* growth (Fig. 1D). Immune cell infiltrates appeared to colocalize with regions of *C. tropicalis* colonization in CARD9^−/−^ kidneys. Notably, only the yeast form of *C. tropicalis* was observed in WT kidneys, whereas both yeast and filamentous hyphae were detected in CARD9^−/−^ kidneys (Fig. 1D), suggesting that an effective immune response limits hyphal formation in vivo.

*C. albicans* induces downstream responses through Dectins and CARD9 signaling. Dectin-1^−/−^ and Dectin-2^−/−^ mice display increased susceptibility to disseminated *C. albicans* infection (17, 39). We found that Dectin-1^−/−^, but not Dectin-2^−/−^, mice were significantly more susceptible to *C. tropicalis* infection than were WT controls (Fig. 1E), although clearly less so than CARD9^−/−^ mice. These data reveal a role for the Dectin-1/CARD9 pathway in protection against disseminated *C. tropicalis* infection.

### Protective immunity against *C. tropicalis* is not mediated through IL-17 signaling

**FIGURE 2.**
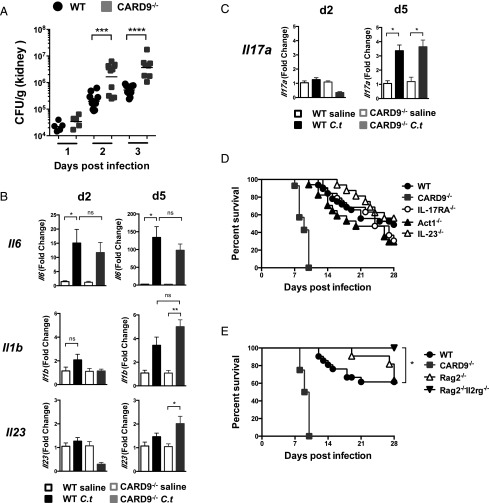
Protective immunity against *C. tropicalis* is not mediated through the IL-17 pathway. (**A**) WT and CARD9^−/−^ kidneys were harvested on the indicated days, and fungal burden was assessed in kidney. Data are pooled from two experiments (each data point represents an individual mouse). ***p < 0.001, ****p < 0.0001, Mann–Whitney *U* test. (**B** and **C**) WT and CARD9^−/−^ kidneys were harvested on the indicated days, and gene expression was assessed by quantitative PCR. Data are representative of two experiments (*n* = 4–10 mice). *p < 0.05, **p < 0.01, Kruskal–Wallis and post hoc Dunn multiple-comparisons tests. (**D**) Survival in the indicated mice was monitored for 28 d. Data are pooled from six experiments (WT, *n* = 32; CARD9^−/−^, *n* = 14; IL-23p19^−/−^, *n* = 16; IL-17RA^−/−^, *n* = 19; Act1^−/−^, *n* = 17). (**E**) Survival in the indicated mice was monitored for 28 d. Data are pooled from four experiments (WT, *n* = 21; CARD9^−/−^, *n* = 16; Rag2^−/−^, *n* = 11; Rag2^−/−^Il2rg^−/−^, *n* = 11). *p < 0.05, log-rank (Mantel–Cox) test. Bar graphs show mean ± SEM. ns, not significant.

One of the major antifungal functions of CARD9 during *C. albicans* infection is believed to be induction of Th17 responses. Splenocytes from CARD9^−/−^ mice infected with *C. albicans* are defective in IL-17A production (13), and both IL-17RA^−/−^ and IL-17A^−/−^ mice display heightened susceptibility to disseminated *C. albicans* infection (14, 15, 17). Based on this model of CARD9 function, we hypothesized that the susceptibility of CARD9^−/−^ mice to *C. tropicalis* could be explained by a defective IL-17 response. Accordingly, we measured expression of transcripts associated with IL-17 responses in kidneys of infected WT and CARD9^−/−^ mice. Genes were measured on day 2 postinfection, the time point at which kidney fungal burdens between WT and CARD9^−/−^ mice were beginning to diverge (Fig. 2A). We also assessed gene expression on day 5, when all mice were alive, but weight loss and fungal loads were significantly different. At days 2 and 5, expression of *Il6* was elevated in both infected WT and CARD9^−/−^ mice at similar levels (Fig. 2B). Changes in *Il1b* expression were only noted on day 5 and also did not differ between CARD9^−/−^ and WT mice (Fig. 2B). There was no increase in *Il23* in WT or CARD9^−/−^ kidneys at day 2 postinfection, although a small, but significant, increase in *Il23* expression was detected in infected CARD9^−/−^ mice on day 5 (Fig. 2B). In line with increased expression of IL-17–inducing cytokine genes, *Il17a* was induced similarly in *C. tropicalis*–infected WT and CARD9^−/−^ mice at day 5 (Fig. 2C), indicating that CARD9^−/−^ mice do not show defective IL-17 expression during *C. tropicalis* infection. Therefore, expression of Th17-inductive cytokines or IL-17 did not correlate with susceptibility to disease.

To determine more directly whether the IL-17 pathway protects from infection, mice lacking components of the IL-17 pathway were subjected to *C. tropicalis* infection. Strikingly, no difference in susceptibility to *C. tropicalis* was detected between WT mice and IL-23p19^−/−^, IL-17RA^−/−^, or Act1^−/−^ mice, in contrast with CARD9^−/−^ controls (Fig. 2D). We next asked whether susceptibility was dependent on cells bearing rearranged AgRs by infecting Rag2^−/−^ mice (lacking T, B, and NKT cells) or Rag2^−/−^Il2rg^−/−^ mice (lacking T, B, NKT, NK, and innate lymphoid cells). Strikingly, neither Rag2^−/−^ nor Rag2^−/−^Il2rg^−/−^ mice displayed increased susceptibility to infection (Fig. 2E). Thus, CARD9-dependent protection against *C. tropicalis* is independent of its effects on the IL-17 pathway or any cell requiring a rearranged AgR.

### Neutrophils and monocytes are required for protection against disseminated *C. tropicalis* infection

**FIGURE 3.**
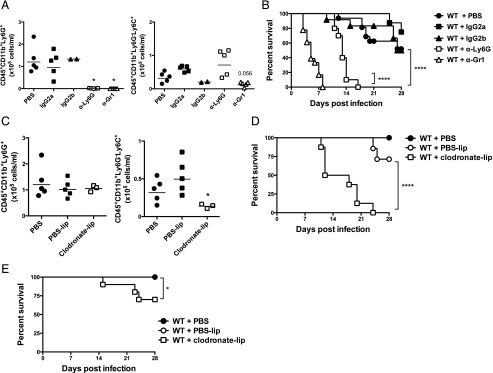
Neutrophils and monocytes are required for protection against disseminated *C. tropicalis*. (**A** and **B**) WT mice were treated with anti-Ly6G or anti-Gr1 Ab or isotype-control Abs on days −1 and +1 and infected with 1 × 10^4^ CFU/g *C. tropicalis* yeast cells on day 0. (A) CD45^+^CD11b^+^Ly6G^+^ neutrophil and CD45^+^CD11b^+^Ly6G^−^Ly6C^+^ monocyte numbers were measured by flow cytometry of blood samples at the time of *C. tropicalis* infection. Data are representative of two experiments (each data point represents an individual mouse). Cells were gated through leukocyte, single cell, and live cell gates. *p < 0.05, Mann–Whitney *U* test. (B) Survival of each cohort was monitored for 28 d. Data are pooled from six experiments (WT + PBS, *n* = 16; WT + IgG2a, *n* = 8; WT + IgG2b, *n* = 12; WT + anti-Ly6G, *n* = 10; WT + anti-Gr1, *n* = 18). ****p < 0.0001, log-rank (Mantel–Cox) test. (**C** and **D**) WT mice were treated with PBS, PBS liposomes (PBS-lip), or clodronate liposomes (Clodronate-lip) 24 h prior to infection with *C. tropicalis* yeast cells on day 0. (C) CD45^+^CD11b^+^Ly6G^+^ neutrophil and CD45^+^CD11b^+^Ly6G^−^Ly6C^+^ monocyte numbers were measured by flow cytometry of blood samples at the time of *C. tropicalis* infection. Data are representative of two experiments (each data point represents an individual mouse). Cells were gated through leukocyte, single cell, and live cell gates. *p < 0.05, Mann–Whitney *U* test. (D) Survival of each cohort was monitored for 28 d. Data are pooled from two experiments (WT + PBS, *n* = 5; WT + PBS-lip, *n* = 7; WT + clodronate-lip, *n* = 8). ****p < 0.0001, log-rank (Mantel–Cox) test. (**E**) WT mice were treated with PBS, PBS-lip, or clodronate-lip 3 d prior to infection with *C. tropicalis* yeast cells on day 0. Survival of each cohort was monitored for 28 d. Data are pooled from two experiments (WT + PBS, *n* = 10; WT + PBS-lip, *n* = 9; WT + clodronate-lip, *n* = 10). *p < 0.05, log-rank (Mantel–Cox) test.

These results pointed to myeloid cells as likely mediators of immunity to *C. tropicalis*. Consistent with this idea is the fact that CARD9 is primarily expressed in myeloid lineage cells (12). Furthermore, neutropenia is a risk factor for disseminated *C. tropicalis* infection in humans, and mice deficient in neutrophils or monocytes/macrophages display increased susceptibility to disseminated *C. albicans* infection (25, 40–43). Accordingly, we first investigated a role for neutrophils in immunity to *C. tropicalis* by treating WT mice with anti-Ly6G Abs to deplete this population (Fig. 3A). Mice were given Abs on days −1 and +1 postinfection, and survival following *C. tropicalis* infection was assessed. As predicted, WT mice given anti-Ly6G Abs were significantly more susceptible to disseminated *C. tropicalis* infection compared with controls (Fig. 3B). All anti-Ly6G Ab–treated mice succumbed to infection by day 17 in contrast to only ∼10% mortality observed in controls at this time point (Fig. 3B). We also treated WT mice with anti-Gr1 Abs to deplete both neutrophils and monocytes (Fig. 3A). Anti-Gr1 Ab–treated mice were even more susceptible to *C. tropicalis* than were anti-Ly6G Ab–treated mice, with 100% mortality by day 8 (Fig. 3B). These results suggest that neutrophils are crucial for protective responses to *C. tropicalis*. Moreover, depletion of both neutrophils and monocytes leads to further enhanced susceptibility to disseminated *C. tropicalis* infection compared with depletion of neutrophils alone.

The increased susceptibility of anti-Gr1 Ab–treated mice compared with anti-Ly6G Ab–treated mice (Fig. 3B) indicated that monocytes, in collaboration with neutrophils, were required for optimal protection against *C. tropicalis* infection. However, the enhanced susceptibility of anti-Gr1 Ab–treated mice could be due to differences in efficacy of neutrophil depletion (44). To confirm a role for monocytes, we injected WT mice i.v. with clodronate liposomes 1 d prior to *C. tropicalis* infection to deplete monocytes but not neutrophils (Fig. 3C) (43, 45). Mice treated with clodronate liposomes were significantly more susceptible to infection compared with controls, with 100% mortality observed at day 24 (Fig. 3D). These data provide further evidence that monocytes are critically involved in protection against disseminated *C. tropicalis* infection.

Notably, a caveat of clodronate treatment is that tissue-resident macrophages, as well as monocytes, may be depleted (46). Therefore, to distinguish the contribution of monocytes and macrophages, we injected clodronate liposomes 3 d prior to *C. tropicalis* infection. With this depletion strategy, monocytes recover to baseline levels, whereas tissue-resident macrophages remain depleted at the time of *C. tropicalis* infection (43, 45). WT mice treated with clodronate liposomes were significantly more susceptible than controls (Fig. 3E); however, the majority of mice survived to 28 d, in contrast to mice given clodronate liposomes 24 h prior to *C. tropicalis* infection (Fig. 3D). Therefore, monocytes, rather than tissue-resident macrophages, play a dominant role in host defense against *C. tropicalis*.

### TNF-α responses are required for protection against *C. tropicalis* and are CARD9 dependent

**FIGURE 4.**
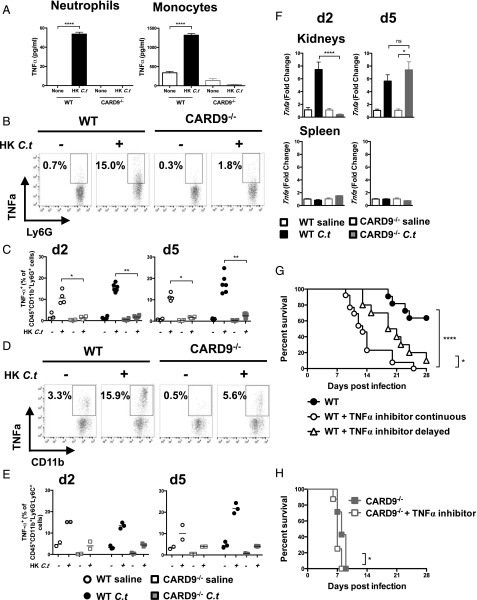
TNF-α responses are impaired in CARD9^−/−^ mice and are required for protection against *C. tropicalis* infection. (**A**) WT or CARD9^−/−^ neutrophils or monocytes were isolated from naive bone marrow and treated or not with HK *C.t* for 24 h in vitro. TNF-α in supernatants was measured by ELISA. Data are representative of two or three experiments (*n* = 2/group). Bar graphs show mean ± SEM. ****p < 0.0001, unpaired Student *t* test. (**B**–**E**) Mice were infected with 1 × 10^4^ CFU/g *C. tropicalis* yeast cells, and blood was harvested on the indicated days. Cells were cultured or not with HK *C.t* for 3 h, and TNF-α produced by CD45^+^CD11b^+^Ly6G^+^ neutrophils (B and C) and CD45^+^CD11b^+^Ly6G^−^Ly6C^+^ monocytes (D and E) was measured by flow cytometry. Data are pooled from (C) or are representative of (E) two experiments (each data point represents an individual mouse). Cells were gated through leukocyte, single cell, and live cell gates. *p < 0.05, **p < 0.01, Mann-Whitney *U* test. (**F**) Kidneys and spleens were harvested on the indicated days, and gene expression was assessed by quantitative PCR. Data are representative of two experiments (*n* = 4–10 mice). Bar graphs show mean ± SEM. *p < 0.05, ****p < 0.0001, Kruskal–Wallis and post hoc Dunn multiple-comparisons tests. (**G**) WT mice were treated with TNF-α inhibitor (etanercept) every 2 d starting on day −1 (“continuous”) or on day 2 or 5 (“delayed”). Data are pooled from two or three experiments (WT, *n* = 11; WT + TNF-α inhibitor continuous, *n* = 13; WT + TNF-α inhibitor delayed, *n* = 10). (**H**) CARD9^−/−^ mice were treated or not with etanercept every 2 d starting on day −1 postinfection. Data are pooled from two experiments (CARD9^−/−^ *n* = 7, CARD9^−/−^ + TNF-α inhibitor, *n* = 8). For (G) and (H), *p < 0.05, ****p < 0.0001, log-rank (Mantel–Cox) test. ns, not significant.

TNF-α is produced rapidly upon disseminated *C. albicans* infection and is required for host defense (47, 48). Monocytes are prominent producers of TNF-α, although this cytokine can potentially be produced by many cell types, including neutrophils (49). Given the rapid mortality of infected CARD9^−/−^ mice and the requirement for neutrophils and monocytes in this setting, we hypothesized that TNF-α production by these cell types is important for host defense against *C. tropicalis*. To test this hypothesis, we first measured TNF-α production by WT and CARD9^−/−^ bone marrow neutrophils and monocytes that were stimulated with HK *C.t* in vitro. Strikingly, CARD9^−/−^ neutrophils and monocytes were completely defective in TNF-α production following HK *C.t* stimulation (Fig. 4A). To determine whether CARD9^−/−^ neutrophils and monocytes were impaired in TNF-α production during *C. tropicalis* infection, we assessed TNF-α levels in the blood of WT and CARD9^−/−^ mice by flow cytometry on days 2 and 5. In line with in vitro data, both neutrophils and monocytes isolated from the blood of CARD9^−/−^ mice on days 2 and 5 were severely impaired in TNF-α production in response to HK *C.t* compared with WT mice (Fig. 4B–E). Thus, CARD9 mediates the production of TNF-α by neutrophils and monocytes during disseminated *C. tropicalis* infection.

To investigate whether TNF-α responses were impaired in kidneys of CARD9^−/−^ mice, we evaluated *Tnfa* expression by quantitative PCR in WT and CARD9^−/−^ mice during infection. *Tnfa* was increased in kidneys of *C. tropicalis*–infected WT mice at day 2 and was maintained at day 5 (Fig. 4F). Strikingly, there was no increase in *Tnfa* expression in CARD9^−/−^ kidneys at day 2 (Fig. 4F). However, by day 5, *Tnfa* was detected in CARD9^−/−^ kidneys, with levels equivalent to WT mice (Fig. 4F), indicating that TNF-α production is transiently impaired in the kidneys of CARD9^−/−^ mice during the early stages of *C. tropicalis* infection. Because we did not detect differences in splenic fungal burdens in WT and CARD9^−/−^ mice (Fig. 1B), we reasoned that TNF-α levels in this organ might be normal in CARD9^−/−^ mice. However, we did not detect *Tnfa* expression in the spleens of either WT or CARD9^−/−^ mice during infection (Fig. 4F), highlighting differences in antifungal immunity between the spleen and kidneys. Overall, these data demonstrate that TNF-α production was strongly impaired in CARD9^−/−^ mice.

To determine whether TNF-α is required for protection against *C. tropicalis* in vivo, we treated WT mice with a soluble TNFR-blocking agent (etanercept), starting at day −1 and continuing every 2 d throughout the infection (continuous). WT mice treated with etanercept were markedly more susceptible to *C. tropicalis* than controls (Fig. 4G), confirming a protective role for TNF-α. The early transient defect in TNF-α expression in CARD9^−/−^ mice suggested that rapid production of this cytokine during *C. tropicalis* infection is crucial for protection. To determine whether early induction of TNF-α was required for immunity to *C. tropicalis*, we assessed survival in WT mice in which etanercept treatment was not started until day 2 postinfection (delayed), meaning that TNF-α responses before this time point would be intact. Delayed treatment with TNF-α inhibitor also increased susceptibility to *C. tropicalis* infection compared with WT mice (Fig. 4G), but not as dramatically as treatment at early time points. Thus, early TNF-α signaling is needed to orchestrate effective immunity to *C. tropicalis*.

Although CARD9 signaling induces TNF-α, other pathways are capable of inducing this cytokine. Indeed, impaired *Tnfa* mRNA expression in CARD9^−/−^ neutrophils was only seen transiently during *C. tropicalis* infection; by day 5 there was no difference compared with WT mice (Fig. 4F). To determine whether there are additional TNF-α–inducing stimuli that contribute to *C. tropicalis* immunity in vivo, TNF-α was depleted in CARD9^−/−^ mice, and survival was compared with control-treated CARD9^−/−^ mice. As shown, CARD9^−/−^ mice administered etanercept were only slightly (albeit significantly) more susceptible to infection compared with controls (Fig. 4H). Thus, there may be CARD9-independent mechanisms that drive TNF-α production, but the majority of the response seems to rely on CARD9. Cumulatively, these results demonstrate a crucial role for CARD9-dependent TNF-α function in host defense against *C. tropicalis* infection.

### CARD9-induced TNF-α is not required for neutrophil and monocyte expansion or recruitment

**FIGURE 5.**
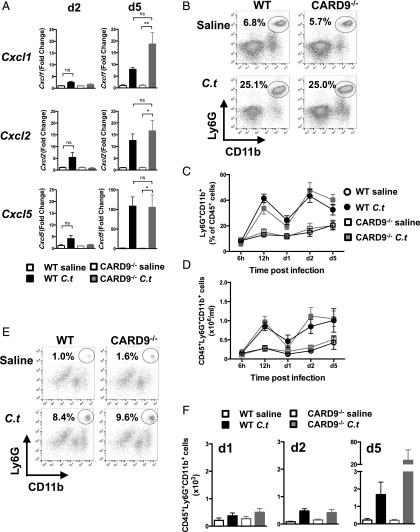
Neutrophil expansion and recruitment to the kidneys are not impaired in CARD9^−/−^ mice during *C. tropicalis* infection. (**A**) WT and CARD9^−/−^ kidneys were harvested on the indicated days, and gene expression was assessed by quantitative PCR. Data are representative of two experiments (*n* = 4–10 mice). Bar graphs show mean ± SEM. Blood (**B**–**D**) and kidneys (**E** and **F**) were analyzed by flow cytometry for CD45^+^CD11b^+^Ly6G^+^ neutrophils at the indicated time points postinfection. Data were pooled from two to six experiments (*n* = 4–20 mice/time point). Cells were gated through leukocyte, single cell, live cell, and CD45^+^ gates. *p < 0.05, **p < 0.01, Kruskal–Wallis and post hoc Dunn multiple-comparisons tests.

TNF-α can exert multiple effects on neutrophils and monocytes. For example, TNF-α acts upon nonhematopoietic cells to induce production of chemokines that recruit neutrophils, such as CXCL1, CXCL2, and CXCL5 (50–53). In this regard, defective neutrophil recruitment in CARD9^−/−^ mice was demonstrated in *Aspergillus fumigatus* infection (54, 55). Additionally, TNF-α can signal directly in neutrophils and monocytes to enhance killing of *C. albicans* and other fungi (56, 57). To evaluate neutrophil activity during *C. tropicalis* infection, we measured expression of neutrophil-attracting chemokines in kidneys of infected mice. Expression of *Cxcl1*, *Cxcl2*, and *Cxcl5* was not detected until day 5 postinfection but was similar between WT and CARD9^−/−^ mice (Fig. 5A). These results hinted that recruitment of neutrophils was unlikely to be impaired in *C. tropicalis*–infected CARD9^−/−^ mice. To confirm this hypothesis, we measured the expansion and recruitment of neutrophils in the blood and kidneys during infection. An increase in both the percentage and absolute numbers of neutrophils was observed at 12 h in blood of infected WT and CARD9^−/−^ mice (Fig. 5C, 5D). A decrease in neutrophils at day 1 was followed by a second wave of expansion at day 2 in both WT and CARD9^−/−^ mice (Fig. 5B–D). At day 5, neutrophil levels remained unchanged. In the kidneys, no detectable difference in neutrophil numbers was seen at day 1 postinfection (Fig. 5F). However, an increase in neutrophils was detected at day 2 in both infected WT and CARD9^−/−^ mice, and neutrophils were further expanded at day 5 (Fig. 5E, 5F). Notably, neutrophil numbers in the kidneys of CARD9^−/−^ mice exceeded those in WT kidneys at day 5, suggesting overzealous neutrophil responses in CARD9^−/−^ mice at this later time point.

**FIGURE 6.**
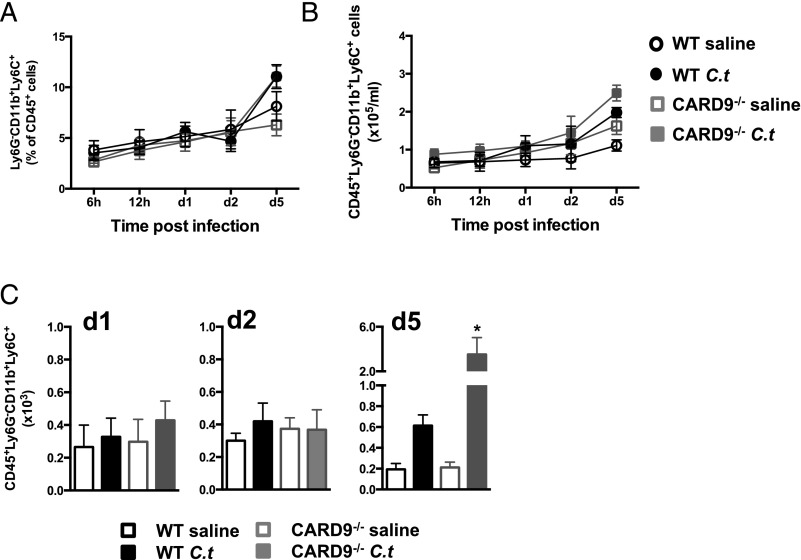
Monocyte expansion and recruitment to the kidneys is not impaired in CARD9^−/−^ mice during *C. tropicalis* infection. Blood (**A** and **B**) and kidneys (**C**) were analyzed by flow cytometry for CD45^+^CD11b^+^Ly6G^−^Ly6C^+^ monocytes at the indicated time points postinfection. Data are pooled from two to six experiments (*n* = 4–20 mice/time point). Cells were gated through leukocyte, single cell, live cell, and CD45^+^ gates. *p < 0.05, Kruskal-Wallis and post hoc Dunn multiple-comparisons tests.

We next assessed the expansion and recruitment of monocytes in the blood and kidneys of *C. tropicalis*–infected mice. In contrast to neutrophil numbers, monocyte expansion in the bloodstream was not detected until day 5 in both WT and CARD9^−/−^ mice (Fig. 6A, 6B). Similarly, an increase in monocyte numbers was only detected in the kidneys of WT and CARD9^−/−^ mice at day 5 (Fig. 6C). No defect in monocyte expansion or recruitment was detected in the blood or kidneys in CARD9^−/−^ mice. In fact, monocyte absolute numbers were markedly higher in the kidneys of CARD9^−/−^ mice compared with WT mice at day 5, in line with elevated neutrophil responses (Figs. 5F, 6C). Together, these data demonstrate that CARD9^−/−^ mice are not impaired in neutrophil and monocyte expansion and recruitment during *C. tropicalis* infection. Rather, CARD9 deficiency leads to heightened neutrophil and monocyte responses in the kidneys that may contribute to pathogenicity.

### CARD9-induced TNF-α is important for neutrophil, but not monocyte, killing

**FIGURE 7.**
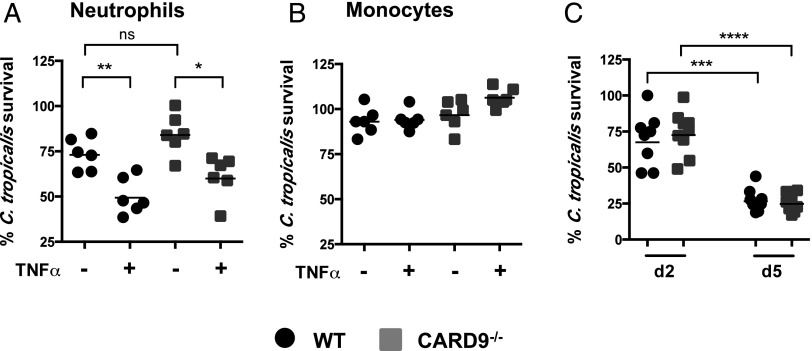
TNF-α enhances *C. tropicalis* killing by neutrophils but not monocytes. WT or CARD9^−/−^ neutrophils (**A**) or monocytes (**B**) were isolated from naive bone marrow and treated or not with TNF-α, followed by incubation with *C. tropicalis* for 2 h. Killing ability was assessed by measuring CFU from neutrophil: *C. tropicalis* cultures or monocyte: *C. tropicalis* cultures. (**C**) Neutrophils were isolated from the blood of WT or CARD9^−/−^ mice infected with *C. tropicalis* for 2 or 5 d. Neutrophil killing ability was assessed by measuring CFU from neutrophil:*C. tropicalis* cultures. Data are pooled from two to four experiments (each data point represents an individual mouse). *p < 0.05, **p < 0.01, ***p < 0.001, ****p < 0.0001, Mann–Whitney *U* test. ns, not significant.

TNF-α enhances neutrophil killing of *C. albicans* and *C. glabrata* (56, 58), but its effects on *C. tropicalis* have not been investigated. To test the hypothesis that CARD9-induced TNF-α augments neutrophil killing of *C. tropicalis*, bone marrow–derived neutrophils from WT or CARD9^−/−^ mice were incubated with this fungus in the presence or absence of TNF-α, and the neutrophil-killing capacity of *C. tropicalis* was determined by measuring CFU. Incubation with TNF-α significantly enhanced the killing of *C. tropicalis* by both WT and CARD9^−/−^ neutrophils (Fig. 7A), demonstrating that TNF-α augments neutrophil killing of *C. tropicalis*. Furthermore, WT and CARD9^−/−^ neutrophils killed *C. tropicalis* equivalently (Fig. 7A). Thus, CARD9^−/−^ neutrophils are not intrinsically defective in *C. tropicalis* killing and can respond to exogenous TNF-α to augment their killing activity.

We next investigated whether TNF-α similarly enhances monocyte killing of *C. tropicalis*. Bone marrow–derived monocytes from WT or CARD9^−/−^ mice were incubated with *C. tropicalis* in the presence or absence of TNF-α, and the killing capacity of monocytes was assessed. In contrast to the effect of TNF-α on neutrophil killing, TNF-α did not enhance WT or CARD9^−/−^ monocyte killing of *C. tropicalis* (Fig. 7B). Moreover, no difference in killing was detected between WT and CARD9^−/−^ monocytes, with both displaying weak killing ability of *C. tropicalis*. Given the positive effect of TNF-α on neutrophil killing in vitro (Fig. 7A) and the impaired TNF-α production in CARD9^−/−^ mice in vivo (Fig. 4A–F), we hypothesized that neutrophils isolated from *C. tropicalis*–infected CARD9^−/−^ mice would be defective in killing the fungus. WT and CARD9^−/−^ neutrophils were isolated from the blood of infected mice on days 2 and 5 and incubated with *C. tropicalis,* and the survival of fungi was measured by CFU enumeration. CARD9^−/−^ neutrophil killing of *C. tropicalis* was equivalent to that of WT neutrophils at both time points (Fig. 7C). Interestingly, WT and CARD9^−/−^ neutrophils isolated from the blood on day 5 were significantly more efficient at killing *C. tropicalis* than were neutrophils isolated on day 2 (Fig. 7C). The data suggest that, at least in vitro, neutrophils isolated from the blood of infected CARD9^−/−^ mice are not impaired in *C. tropicalis* killing.

Taken together, these results indicate that exposure to TNF-α augments the ability of neutrophils, but not monocytes, to kill *C. tropicalis*. Moreover, an environment with reduced TNF-α expression due to impaired production of this cytokine by neutrophils and monocytes dramatically enhances susceptibility to *C. tropicalis.*

## Discussion

Studies of *C. albicans* created a framework for understanding immunity to fungi (4, 59), leading to the prevailing paradigm that Dectin-1 recognition of *C. albicans* leads to CARD9-mediated skewing of T cell responses to a Th17 phenotype, which is essential for fungal clearance (60). Comparatively little is known about immunity toward non-*albicans* fungal species. In this article, we demonstrate that CARD9 is crucial for protection against disseminated *C. tropicalis* infection and that TNF, and not IL-17, is the key antifungal cytokine.

Considerable data show that CARD9 activity extends beyond IL-17. The first report demonstrating the role for CARD9 in protection against disseminated *C. albicans* infection documented 100% mortality of *C. albicans*–infected CARD9^−/−^ mice within 5 d (6). In contrast, at a similar infective dose, 100% mortality of IL-17RA^−/−^ mice was not observed until 14–28 d in two independent studies (14, 15). IL-17 signaling is essential for immunity in oral candidiasis, whereas CARD9 is largely dispensable (61). Moreover, normal IL-17 responses are seen in CARD9-deficient patients with invasive candidiasis (8, 9). Thus, the susceptibility of CARD9^−/−^ mice is not fully explained by defective IL-17 responses.

The basis for the difference in susceptibility to *C. tropicalis* and *C. albicans* in IL-17RA^−/−^ mice is unclear (14, 15). The explanation is unlikely to relate to infectious dose, because the initial report that IL-17RA^−/−^ mice are susceptible to systemic *C. albicans* infection showed no dose dependence (15). Alternatively, there may be strain differences among *C. tropicalis* species; indeed, the susceptibility of Dectin-1^−/−^ mice to candidiasis varies by strain (62). IL-17A is expressed in kidneys of *C. tropicalis*–infected mice (Fig. 2C), indicating that IL-17 responses are induced, even though dispensable for host defense. IL-17A production in response to *C. dubliniensis*, *C. krusei*, and *C. glabrata* also was documented (63). Whether IL-17 is also dispensable for protection against other NAC species is unknown.

Dectin-1^−/−^ mice were more susceptible to *C. tropicalis* than WT mice (Fig. 1E). Similarly, Dectin-1 controls *C. tropicalis* growth in the gut in a model of dextran sulfate sodium–induced colitis (64). Therefore, Dectin-1 is likely to be a key pattern recognition receptor in control of *C. tropicalis* at both mucosal and visceral sites. Of note, ∼50% of Dectin-1^−/−^ mice survived the 28-d infection period (Fig. 1E). Recognition of *C. albicans* involves several CLRs that signal through CARD9, including Dectin-2 and Dectin-3 (17, 65, 66). The fact that we did not see an increased susceptibility in Dectin-2^−/−^ mice (Fig. 1E) indicates that additional CARD9-dependent signaling receptors may be involved.

Although IL-17 was dispensable for immunity to *C. tropicalis*, TNF-α–dependent immunity was essential and was CARD9 dependent. The finding that CARD9^−/−^ neutrophils and monocytes failed to produce TNF-α after exposure to *C. tropicalis* in vitro and ex vivo suggests that both cell types are sources of TNF-α. WT monocytes produced considerably more TNF-α than did neutrophils upon exposure to *C. tropicalis*, at least on a per-cell basis (Fig. 4A–E), suggesting that monocytes are likely to be a major source of this cytokine in vivo. However, the greater overall frequency of neutrophils during infection means that this cell type is probably also a significant overall source. Although TNF-α production by CARD9^−/−^ neutrophils and monocytes was impaired throughout the course of infection, defective TNF-α responses were only transient in the kidneys of CARD9^−/−^ mice. It is likely that other cell populations contribute to TNF-α production at day 5 and beyond, possibly a compensatory mechanism in CARD9^−/−^ mice. Nonetheless, the delayed TNF-α expression was not sufficient to protect CARD9^−/−^ mice from increased mortality.

These data show that rapid induction of myeloid cell/CARD9/TNF-α–mediated immunity is important for disease outcome. WT mice were more susceptible to *C. tropicalis* infection when TNF-α was continuously depleted compared with delayed TNF-α depletion (Fig. 4G). We previously reported transient neutrophil depletion in *C. albicans*–infected WT mice using anti-Ly6G and anti-Gr1 Abs (44), and depletion with these Abs rendered WT mice highly susceptible to *C. tropicalis* infection (Fig. 3B). Therefore, early neutrophil and monocyte responses are needed to control *C. tropicalis*. In line with our data, neutrophil depletion at the time of infection, but not at later time points, led to increased susceptibility (67). A requirement for innate antifungal immunity during candidiasis in humans is suggested by the fact that patient mortality is increased significantly if antifungal drugs are administered within the first 24 h (68). Therefore, our data fit a model wherein neutrophils and monocytes are the first cell types to recognize *C. tropicalis* by Dectin-1 and additional pattern recognition receptors, leading to the production of TNF-α in response to the fungus in a CARD9-dependent manner. TNF-α then acts primarily upon neutrophils to increase their ability to kill *C. tropicalis*, ultimately controlling disease.

There was no difference in the intrinsic capacity of WT and CARD9^−/−^ neutrophils to kill *C. tropicalis*, contrasting with data in humans. Neutrophils from candidiasis patients with a *CARD9* defect exhibited impaired killing of *C. albicans* (19, 20) and the fungus *Phialophora verrucosa* (69). There may be species-dependent differences in myeloid function, because murine neutrophils show a reduced ability to kill *C. albicans* compared with human neutrophils (70). Mice also harbor a lower frequency of circulating neutrophils than humans. In contrast to neutrophils, CARD9^−/−^ monocytes killed *C. tropicalis* similarly to WT. However, monocytes generally showed poor candidacidal activity, with ∼90% *C. tropicalis* survival upon monocyte coincubation. Human neutrophils are also more efficient at killing *C. tropicalis* than monocytes (71). Moreover, TNF-α did not enhance monocyte killing of *C. tropicalis*; this may be due to the duration of TNF-α exposure, because one study showed that TNF-α augments human monocyte killing of *C. tropicalis* following prestimulation for 24 h but not 2 h (71). Collectively, our data suggest that neutrophils are the key cell population involved in *C. tropicalis* killing.

Given that TNF-α significantly augments neutrophil killing of *C. tropicalis*, it was surprising that CARD9^−/−^ neutrophils isolated from infected blood were not defective in *C. tropicalis* killing compared with WT neutrophils. This could be due to a lack of exposure to TNF-α in the assay used in this study (i.e., in vivo, neutrophils are exposed to TNF-α continually as the result of production by monocytes and other cell types). This TNF-α–rich environment is removed upon isolation of WT neutrophils from *C. tropicalis* infection. We hypothesize that the positive effects of TNF-α on neutrophil killing depend on continued exposure to TNF-α.

Unexpectedly, there was no apparent role for CARD9 in neutrophil recruitment during *C. tropicalis* infection. This finding contrasts with a report documenting a requirement for CARD9 in neutrophil recruitment during *A. fumigatus* infection, which was associated with reduced expression of CXCL1, CXCL2, and CXCL5 (55). However, these chemokines were not altered in CARD9^−/−^ mice during *C. tropicalis* infection. Interestingly, mice deficient in TNF-α and LTα display increased susceptibility to disseminated *C. albicans* infection associated with reduced kidney neutrophil recruitment (72). In this regard, our finding that tissue-resident macrophages do not play a significant role in disseminated *C. tropicalis* contrasts with *C. albicans* (43), highlighting another difference in immunity to these species.

We were intrigued by the increased susceptibility to *C. tropicalis* upon TNF blockade. Etanercept, a soluble TNFR, is widely used to treat autoimmune diseases. One study documented increased susceptibility to oral candidiasis in patients receiving etanercept (73). To our knowledge, there has been no report linking anti–TNF-α agents to invasive *C. tropicalis* infection. However, *C. tropicalis* is most common in countries where there is limited access to expensive biologic therapies, so susceptibility to this organism may not be apparent. Therapies targeting IL-17A were approved recently for clinical use in psoriasis (74). Data from clinical trials documented increased rates of mucosal candidiasis following treatment with secukinumab (anti–IL-17A Ab) (75). Our data raise the possibility that patients taking anti–IL-17 biologic drugs are not likely to have an increased risk for morbidity or mortality to invasive *C. tropicalis* infections.
